# A Smac-mimetic sensitizes prostate cancer cells to TRAIL-induced apoptosis via modulating both IAPs and NF-kappaB

**DOI:** 10.1186/1471-2407-9-392

**Published:** 2009-11-06

**Authors:** Yao Dai, Meilan Liu, Wenhua Tang, Yongming Li, Jiqin Lian, Theodore S Lawrence, Liang Xu

**Affiliations:** 1 Department of Radiation Oncology, University of Michigan, Ann Arbor, MI 48109, USA; 2 Department of Pathology, University of Michigan, Ann Arbor, MI 48109, USA; 3 Comprehensive Cancer Center, University of Michigan, Ann Arbor, MI 48109, USA; 4 Current address : Department of Radiation Oncology, University of Florida Health Science Center, Gainesville, FL 32610

## Abstract

**Background:**

Although tumor necrosis factor-related apoptosis-inducing ligand (TRAIL) is a promising agent for human cancer therapy, prostate cancer still remains resistant to TRAIL. Both X-linked inhibitor of apoptosis (XIAP) and nuclear factor-kappaB function as key negative regulators of TRAIL signaling. In this study, we evaluated the effect of SH122, a small molecule mimetic of the second mitochondria-derived activator of caspases (Smac), on TRAIL-induced apoptosis in prostate cancer cells.

**Methods:**

The potential of Smac-mimetics to bind XIAP or cIAP-1 was examined by pull-down assay. Cytotoxicity of TRAIL and/or Smac-mimetics was determined by a standard cell growth assay. Silencing of XIAP or cIAP-1 was achieved by transient transfection of short hairpin RNA. Apoptosis was detected by Annexin V-PI staining followed by flow cytometry and by Western Blot analysis of caspases, PARP and Bid. NF-kappaB activation was determined by subcellular fractionation, real time RT-PCR and reporter assay.

**Results:**

SH122, but not its inactive analog, binds to XIAP and cIAP-1. SH122 significantly sensitized prostate cancer cells to TRAIL-mediated cell death. Moreover, SH122 enhanced TRAIL-induced apoptosis via both the death receptor and the mitochondrial pathway. Knockdown of both XIAP and cIAP-1 sensitized cellular response to TRAIL. XIAP-knockdown attenuated sensitivity of SH122 to TRAIL-induced cytotoxicity, confirming that XIAP is an important target for IAP-inhibitor-mediated TRAIL sensitization. SH122 also suppressed TRAIL-induced NF-kappaB activation by preventing cytosolic IkappaB-alpha degradation and RelA nuclear translocation, as well as by suppressing NF-kappaB target gene expression.

**Conclusion:**

These results demonstrate that SH122 sensitizes human prostate cancer cells to TRAIL-induced apoptosis by mimicking Smac and blocking both IAPs and NF-kappaB. Modulating IAPs may represent a promising approach to overcoming TRAIL-resistance in human prostate cancer with constitutively active NF-kappaB signaling.

## Background

Primary or acquired resistance of prostate cancer to current treatment protocols has been associated with apoptosis-resistance in cancer cells, leading to therapy failure [1,2]. Tumor necrosis factor-related apoptosis-inducing ligand (TRAIL) is a member of the TNF family that is in clinical trials for the treatment of prostate cancer, either alone or in combination with other treatments [3]. TRAIL selectively induces apoptosis in prostate cancer cells compared to normal prostate epithelial cells [4]. The relative resistance of normal cells to TRAIL has been explained by the lower expression levels of functional death receptors relative to cancer cells [5,6]. Hence, TRAIL exerts a selective antitumor activity without eliciting systemic toxicity in multiple preclinical models, and is considered to be a prime candidate for prostate cancer therapy [3].

Mechanistically, TRAIL triggers apoptosis via binding to its functional death receptors DR4 and DR5, and activating both death receptor (extrinsic) and mitochondria (intrinsic) apoptosis pathways [7]. Ligation of DR4/DR5 by TRAIL results in caspase-8 activation and directly cleaves downstream effector caspases [8]. Signals originating from death receptors can be linked to mitochondria via Bid, which causes mitochondrial cytochrome c release and caspase-9 activation. The mitochondrial pathway is engaged by the release of multiple pro-apoptotic factors from mitochondria into the cytosol, such as cytochrome c, Smac and apoptosis inducing factor (AIF). These factors execute cells through apoptosis in either a caspase-dependent or independent manner [9].

Despite the fact that TRAIL selectively induces apoptosis in cancer cells, TRAIL-resistance has been observed in a substantial number of cancers, including prostate cancer [10]. It is widely accepted that the inhibitor of apoptosis proteins (IAP) function as a key negative regulator in TRAIL resistance [11,12]. Mounting evidence confirms that XIAP, the most potent anti-apoptotic protein among IAPs, is responsible for primary or acquired TRAIL resistance in tumor cells [13-16]. Overexpression of XIAP increases resistance to TRAIL-induced apoptosis, while downregulation of XIAP restores responsiveness to TRAIL [17,18]. At the transcriptional level, almost all IAP proteins are driven by the upstream transcription factor NF-kappa B (NF-κB), which can be stimulated by multiple stimuli, including TRAIL [19]. TRAIL-induced NF-κB activation attenuates apoptosis, predominantly by upregulating various anti-apoptotic proteins, including IAPs [20,21]. Therefore, NF-κB functions as an upstream regulator of IAPs and negatively regulates TRAIL signaling. The role of NF-κB in the anti-apoptotic process has been studied in prostate cancer cells both *in vitro *and *in vivo*. In prostate cancer cell lines, there seems to be an inverse correlation between androgen receptor (AR) status and constitutive NF-κB activity [22]. Thus it is tempting to speculate that the constitutive activation of NF-κB may contribute to prostate cancer cell survival and treatment resistance following androgen ablation [22].

Smac functions as an endogenous IAP-antagonist [23]. Upon stimulation by TRAIL, Smac is released from mitochondria into the cytosol [24]. The released Smac interacts with XIAP through the N-terminal four conserved amino acid residues (AVPI) that bind to the baculoviral IAP repeat 3 (BIR3) domain of XIAP and eliminates the inhibitory effect of XIAP on caspase activation [25-27]. Due to the impressive pro-apoptotic role of Smac, synthetic small molecule Smac-mimicking compounds (Smac-mimetics) are being developed to sensitize apoptosis-resistant cancer cells to various apoptotic stimuli [3,28]. Smac-mimetic IAP-antagonists induce TNFα-dependent apoptosis in several transformed cell lines [29-31], and small molecule Smac-mimetics successfully sensitize TRAIL-induced apoptosis by blocking functions of IAPs in multiple cancer cells [11,16,32,33]. These studies provide a solid foundation for our assertion that mimicking Smac may represent a promising strategy for restoring defective apoptosis signaling triggered by TRAIL in prostate cancer therapy.

Based on the high-resolution experimental 3D structure of Smac in complex with the XIAP BIR3 domain, a group of potent non-peptidic Smac-mimetics, called SH-compounds, were designed that mimic the tetra-peptide at the N-terminal of the Smac protein [34,35]. These cell-permeable compounds show at least 20-fold more potential than the natural Smac peptide to bind to the XIAP BIR3 domain in a cell-free system [34-36]. In the current study, we evaluated the sensitizing potential of one of these compounds, SH122, on TRAIL-mediated cell death in several human prostate cancer cell lines. We also investigated potential molecular targets and the mechanism of action involved in SH122-mediated TRAIL sensitization.

## Methods

### Cell Culture and Reagents

Human prostate cancer DU145 and LNCaP cells were obtained from the American Type Culture Collection (Manassas, VA). Androgen-independent prostate cancer cell line CL1 derived from its androgen-dependent cell line LNCaP was kindly provided by Dr. Arie Belldegrun (University of California, Los Angeles). Cells were routinely maintained in Dulbecco's Modified Eagle Medium (DMEM, Gibco) with 10% fetal bovine serum (FBS) and 2 mM L-glutamine. Cultures were maintained in a humidified incubator at 37°C with 5% CO_2_. Small molecule Smac-mimetic SH122 as well as its inactive analogs SH123 and SH110 were kindly provided by Dr. Shaomeng Wang, University of Michigan. TRAIL was purchased from Cell Sciences (Canton, MA). Antibodies against XIAP and cIAP-1 were purchased from BD Biosciences (San Jose, CA). Antibodies against PARP, RelA and IκBα were purchased from Santa Cruz Biotechnology (Santa Cruz, CA). Other antibodies include: anti-caspase-3 (BioVision, Mountain View, CA), anti-caspase-8 (Calbiochem, San Diego, CA), anti-caspase-9 (Novus Biologicals, Littleton, CO), anti-Bid (Cell Signaling Technology, Beverly, MA), and anti-β-actin (Sigma, MO). Chemicals were from Sigma unless otherwise indicated.

### Cytotoxicity Assay

To detect the survival of cells after treatment, a standard cell growth assay was performed using the CCK-8 detection kit (Dojindo Molecular Technologies, Gaithersburg, MD) following the manufacturer's instructions. Absorbance was detected at 450 nm and 650 nm respectively, using a microplate reader (TECAN ULTRA, Research Triangle Park, NC). Cell viability (%) was normalized by dividing normalized absorbance of treated samples by that of the untreated control [37]. Inhibitory concentration 50% (IC_50_) was calculated by GraphPad Prism 5.0 (San Diego, CA).

### Pull-down Assay

Cells (1.0 × 10^7^) were disrupted in a lysis buffer (50 mM Tris-HCl, pH 7.5, 150 mM NaCl, 1% Nonidet P-40, 0.5% sodium deoxycholate), with freshly added protease inhibitor cocktail (Roche). Cell lysates were homogenized by passing through a 27-1/2G syringe needle (BD). After centrifugation at 10,000 × g for 15 min at 4°C, the supernatant was harvested and incubated with biotin-labeled Smac-mimetic compounds with or without non-labeled compounds, for 1 hour at 4°C. Lysates were incubated with pre-cleared Streptavidin-Agarose beads (Invitrogen) by gently rotating for 2 hours at 4°C. The beads were collected and washed with washing buffer (Roche) and eluted in 60 μl of loading buffer (Bio-Rad, Hercules, CA). After boiling for 5 min, the eluents were analyzed by immunoblotting to detect proteins that interacted with the compounds [38].

### Apoptosis Assay

For apoptosis analysis by flow cytometry, DU145 cells were treated with SH122 and TRAIL, alone or in combination, with SH110 used as a negative control. Cells were harvested by trypsinization and washed twice with ice-cold PBS. After centrifuge, cells were stained with 100 μl Annexin V-FITC diluted in binding buffer (10 mM HEPES,100 mM NaCl, 10 mM KCl, 1 mM MgCl_2_, 1.8 mM CaCl_2_) containing propidium iodide (50 μg/ml). Cells were incubated for 15 min at room temperature before analysis by flow cytometry with FACScan (BD) using a 488-nm laser line. Data were analyzed using WinMDI 2.8 software (Purdue University Cytometry Laboratories) as described previously [38].

### shRNA Transfection

The shRNA specific for XIAP in plasmid pRS-shXIAP29 was purchased from OriGene (Rockville, MD). The shRNA specific for cIAP-1 in plasmid pGB-shcIAP-1 was purchased from BioVision (Mountain View, CA). shRNA plasmids (2.0 μg) were transfected into DU145 cells using LipofectAMINE 2000 (Invitrogen, Carlsbad, CA), following the manufacturer's instructions. Forty-eight hours after transfection, cells were treated with Smac-mimetic compounds and/or TRAIL and processed for cytotoxicity analysis. Knockdown effect was detected by Western blot.

### Western Blot Analysis

Whole cell proteins were isolated by RIPA buffer (50 mM Tris-HCl, pH 8.0, 150 mM NaCl, 0.1% SDS, 1% NP-40, 0.25% Sodium deoxycholate and 1 mM EDTA) with freshly added protease inhibitor cocktail (Roche). Whole cell lysates were clarified by centrifugation at 10,000 × g for 10 minutes at 4°C. Total protein concentrations were determined by BCA Protein Assay (Pierce, Rockford, IL). Equal amounts of proteins were loaded to pre-cast 4-20% SDS-PAGE gels (Invitrogen). Proteins resolved on gels were transferred to PVDF membranes (Bio-Rad). After electro-transfer, membranes were blocked with 5% nonfat milk in TBS-T buffer (20 mM Tris-HCl, pH 8.0, 150 mM NaCl, 0.05% Tween 20), and probed with the desired primary antibodies. Blots were then incubated with horseradish-conjugated secondary antibodies and detected with the SuperSignal West Pico chemiluminescence substrate (Pierce), and exposed to an X-ray film (Kodak, Rochester, NY). Intensity of the desired bands was analyzed using TotalLab software (Nonlinear Dynamics, Durham, NC).

### Cytosol and Nuclei Fractionation

To detect subcellular redistribution of NF-κB proteins, cytoplasmic and nuclear fractions were prepared according to the method reported [39] with modifications. Briefly, treated cells (5 × 10^6 ^cells) were resuspended in 200 μl of hypotonic buffer (10 mM HEPES, 5 mM KCl, 1.5 mM MgCl_2_, 1 mM phenylmethylsulfonyl fluoride (PMSF), 1 mM dithiothreitol (DTT), and protease inhibitors), mixed well, and incubated with constant rotation at 4°C for 15 min. Nonidet P-40 (10%) was then added to reach a final concentration of 0.5%. Cytosolic extract was cleared by centrifugation at 12,000 rpm for 1 min. The pellets were washed once with hypotonic buffer, and resolved in nucleus extraction buffer (20 mM HEPES, 50 mM KCl, 300 mM NaCl, 1 mM PMSF, 1 mM DTT, and protease inhibitors), with constant rotation at 4°C for 45 min. Nuclear extracts were harvested after centrifuging for 10 min at 12,000 rpm. Subcellular proteins were quantified by BCA assay before being employed to Western blot analysis.

### Quantitative Real-time PCR (qRT-PCR)

qRT-PCR was performed to determine the NF-κB target gene expression level [40]. Total RNA was extracted from DU145 cells using TRIzol (Invitrogen). Reverse transcription reaction with 1 μg of total RNA in 100 μl was carried out following the instructions of the TaqMan Reverse Transcription Kit (Applied Biosystems, Foster City, CA). For quantitative PCR, 1 μl of gene primers with SYBR Green (Applied Biosystems) in 20 μl of reaction volume was applied [41]. Primers were designed as: TNF, forward, 5'CCAGGGACCTCTCTCTAATCAGC3', reverse, 5'CTCAGCTTGAGGGTTTGCTACAA3'; IL8, forward, 5'CGTGGCTCTCTTGGCAGC3', reverse, 5'TCTTTAGCACTCCTTGGCAAAAC3'; Actin (as an internal control): forward, 5'ATGCAGAAGGAGATCACTGC3', reverse, 5'TCATAGTCCGCCTAGAAGCA3'. BIRC4, forward, 5'AGTGGTAGTCCTGTTTCAGCATCA3', reverse, 5'CCGCACGGTATCTCCTTCA3'. FAM probed ICAM-1 primers were commercially obtained from Applied Biosystems. All reactions with TaqMan PCR Master Mix (Applied Biosystems) were performed on the Mastercycler *realplex*^2 ^S (Eppendorf, Westbury, NY). Target gene mRNA levels were normalized to actin mRNA according to the following formula: [2^-(C_T _target - C_T _Actin)] × 100%, where CT is the threshold cycle. Fold increase was calculated by dividing the normalized target gene expression of the treated sample by that of the untreated control [41,42].

### NF-κB Reporter Assay

DU145 cells were seeded into a 48-well plate 24 h before transfection. For each well, cells were transiently cotransfected with 0.1 μg of a NF-κB reporter construct (pNF-κB) or a control reporter plasmid (pControl) (Panomics Inc., Fremont, CA), together with 0.06 μg of β-galactosidase reporter vector (Promega, Madison, WI), which was used to normalize NF-κB reporter gene activity, using LipofectAMINE 2000 (Invitrogen). Twenty-four hours after transfection, cells were treated with TNF-a or TRAIL for 0 h, 1 h, 2 h, 4 h and 6 h. To determine the effect of SH122 on TRAIL-induced NF-κB activation, cells were pretreated with SH122 or SH123 for 1 h followed by TRAIL stimulation for 4 h. Cell lysates were prepared using Reporter Lysis Buffer (Promega). For luciferase and β-galactosidase assays, the samples were measured on a microplate luminometer using the Bright-Glo luciferase assay kit and β-galactosidase enzyme assay kit (Promega), respectively, according to the manufacturer's instructions. Fold of activation was calculated for each treated sample by dividing normalized luciferase activity with that of the untreated control.

### Statistical Analysis

Two-tailed Student's *t*-test was employed, using GraphPad Prism 5.0 software (San Diego, CA). A threshold of *P *< 0.05 was defined as statistically significant.

## Results

### Validation of interactions between Smac-mimetic compound SH122 and IAPs

**Figure 1:**
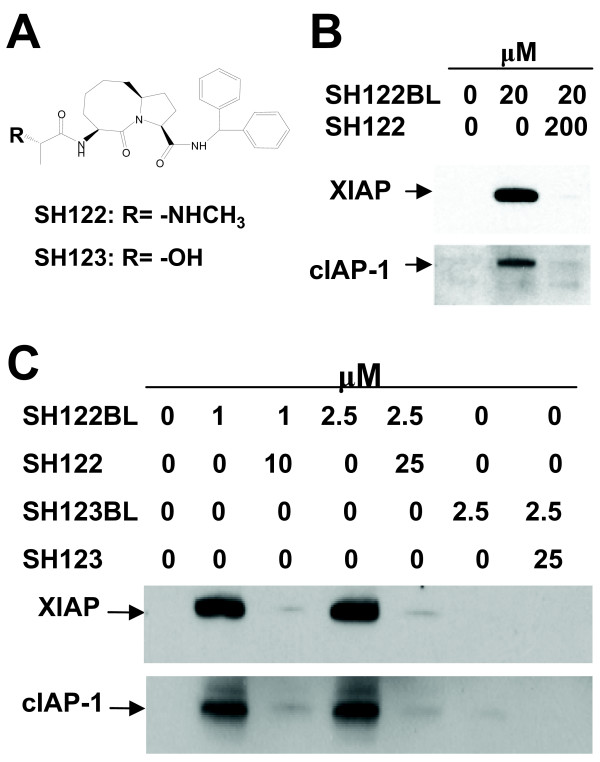
**Smac-mimetic compound interacted with XIAP and cIAP-1 in human prostate cancer cells**. **A**. Structure of the Smac-mimetic compound SH122, and its inactive control compounds SH123 and SH110. **B**. Pull-down assay. Biotin-labeled SH122 (SH122BL, 20 μM) was incubated with whole cell lysates of DU145 cells with or without 10-fold excess of non-labeled SH122, followed by incubation with precleared Streptavidin agarose beads. Eluted beads were employed to Western blot analysis with anti-XIAP or cIAP-1 antibody. Data shown represent one of at least three independent experiments. SH122BL, Biotin-labeled SH122. **C**. CL1 cell lysate was incubated with 1 μM and 2.5 μM of SH122BL, or 2.5 μM of SH-123BL, with or without 10-fold excess of their unlabeled forms. SH123BL, Biotin-labeled SH123.

Based on 3-D rational design and computational modeling, SH122 (Figure 1A) was synthesized and developed as a non-peptide small molecule IAP-antagonist that is 20~30-fold more potent than the N-terminal tetrapeptide of Smac in binding to IAPs, while inactive analogs SH123 and SH110 were shown to be 200-fold less potent [35]. To verify the specific binding of SH122 to IAPs, we employed a pull-down assay with biotin-labeled SH122 (SH122BL) in human prostate cancer cells. As shown in Figure 1B, both XIAP and cIAP-1 were pulled down by SH122 in DU145 cells. Furthermore, pre-incubation with a 10-fold excess of non-biotin-labeled SH122 resulted in a more than 90% block of the binding of SH122BL to XIAP/cIAP-1, suggesting that the binding was specific. In another androgen-independent prostate cancer CL1 cell line, 1 μM of SH122 was sufficient to pull down both XIAP and cIAP-1 (Figure 1C), and as with DU145, the binding effect was blocked by a 10-fold excess of non-labeled SH122. By contrast, the negative control compound SH123BL showed almost no binding to either XIAP or cIAP-1, as evidenced in the previous report [38]. These results demonstrate that Smac-mimetic SH122 potently and specifically interacts with XIAP and cIAP-1 in human prostate cancer cells.

### SH122 sensitizes TRAIL-induced prostate cancer cell growth inhibition

**Figure 2:**
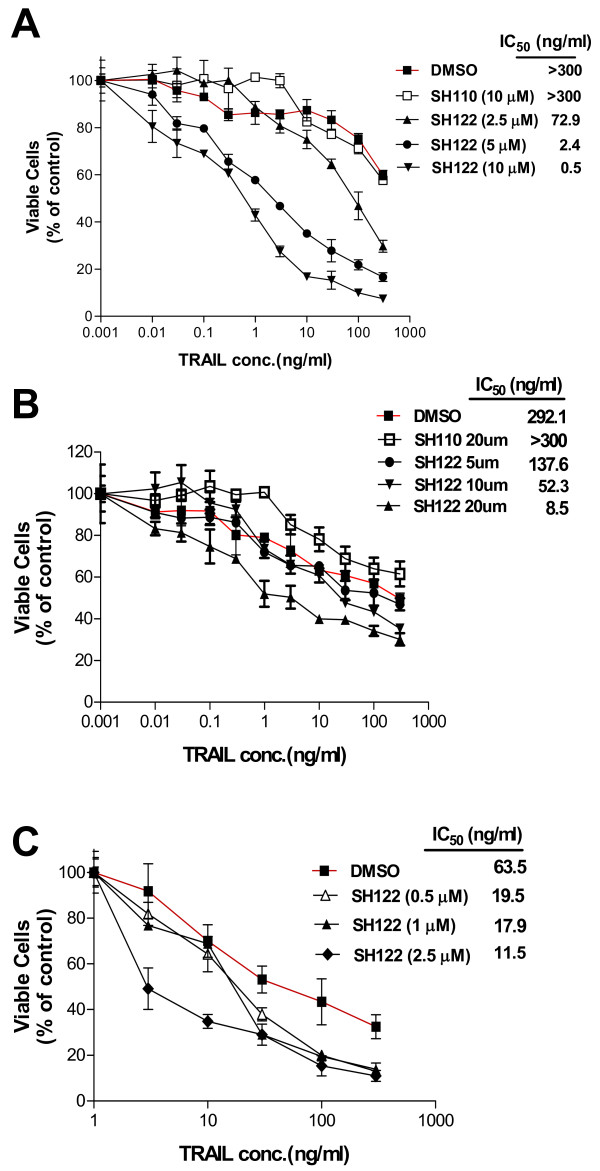
**SH122 promoted TRAIL-induced cell death in prostate cancer cell line DU145 (A), LNCaP (B) and CL1 (C)**. Cells (5,000 cells/well in 96-well plates) were treated with different concentrations of TRAIL and SH122, alone or in combination, with SH110 as a negative control. After incubation for 96 h, cells were stained with Cell Counting kit-8 reagent. The optical density of each sample was measured. Data were normalized as described in *Materials and Methods*. Data were presented as mean ± SD (*n *= 3).

To detect the combination effects of a Smac-mimetic and TRAIL, cytotoxicity was tested after concurrent treatment with TRAIL and SH122. In DU145 cells, while TRAIL alone had a minor effect on decreasing cell viability, 5 μM and 10 μM of SH122 showed a 100- and 600-fold sensitization, respectively, compared to TRAIL alone (Figure 2A). By contrast, the negative control compound SH110 produced no sensitization. Similar results were observed in two other prostate cancer cell lines. As shown in Figure 2B, SH122, but not SH110, potentiated TRAIL-induced cell growth inhibition in LNCaP cells. In CL1 cells that were derived from LNCaP, SH122 showed dose-dependent effects on TRAIL sensitization, although the concentration used was approximately 10-fold less than that for LNCaP cells (Figure 2C). Notably, CL1 seemed to be more sensitive to TRAIL compared with LNCaP, as shown by IC_50_, suggesting that ablation of the androgen-receptor may result in upregulation of TRAIL receptor expression. These data demonstrate that the Smac-mimetic compound SH122 potentiates TRAIL-mediated cytotoxicity in both TRAIL-resistant and TRAIL-sensitive prostate cancer cell lines.

### SH122 enhances TRAIL-induced apoptosis

**Figure 3:**
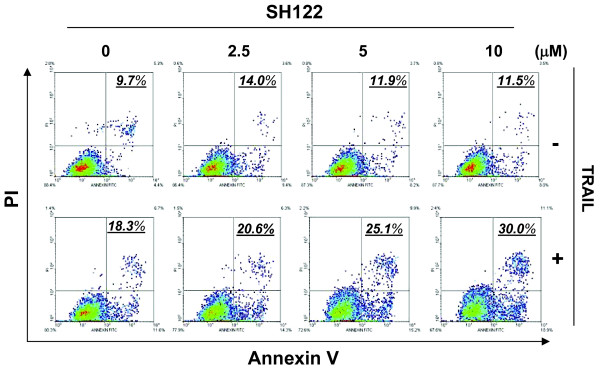
**SH122 enhanced TRAIL-induced apoptosis**. DU145 cells were seeded into 6-well plates at a concentration of 2 × 10^5^/ml, and exposed by 2.5, 5 and 10 μM of SH122, with or without TRAIL (50 ng/ml). Eighteen hours after incubation, cells were harvested and processed for Annexin V-FITC and PI staining by flow cytometry. Numbers represented total apoptosis (Annexin V positive cell population). Data represented one of three independent experiments.

To determine whether apoptosis was involved in TRAIL-induced cell death, we examined apoptosis induced by TRAIL in combination with SH122, with Annexin V-FITC and PI staining by flow cytometry analysis. As shown in Figure 3, SH122 increased TRAIL-induced apoptosis in a dose-dependent manner. Even at a lower concentration (2.5 μM), SH122 enhanced TRAIL-induced total apoptosis as compared with TRAIL and SH122 alone (Figure 3). In contrast, TRAIL alone (50 ng/ml) moderately induced apoptosis, and SH122 alone showed a minor effect on apoptosis, with a slightly increased apoptotic cell population compared with the control even at the highest concentration (Figure 3).

### Both death receptor and mitochondrial pathways are involved in SH122-sensitized, TRAIL-induced apoptosis

**Figure 4:**
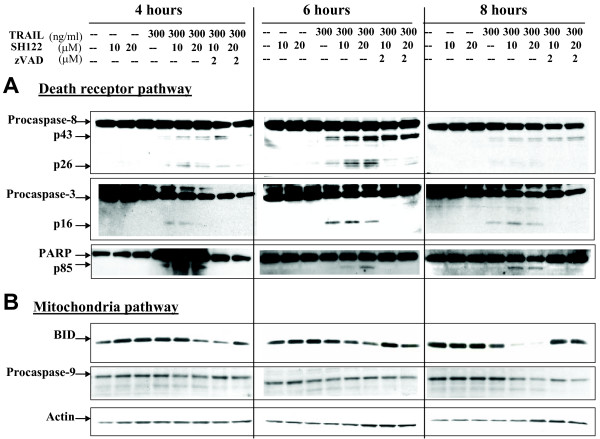
**SH122 potentiated TRAIL-induced apoptosis by activating both the death-receptor pathway (A) and mitochondrial pathway (B)**. DU145 cells were treated with 10 and 20 μM of SH122, in the presence or absence of 300 ng/ml of TRAIL, with or without pretreatment with zVAD (2 μM), for 4, 6, and 8 h, respectively. Whole cell lysates (30 μg) were subjected to Western blot analysis. Membranes were probed with antibodies against caspase-8, caspase-3, caspase-9, PARP and Bid. Actin was shown as a loading control.

Based on TRAIL-induced apoptotic signaling, we examined the expression of caspases treated with SH122 and TRAIL. Caspase-8 was cleaved into fragments (p43/41 and p18) as early as 4 h after exposure to TRAIL alone, while cleavage of caspase-8 became more intense with increased concentrations of SH122, especially at 6 h after treatment (Figure 4A). A similar tendency was observed for caspase-3. Activation of caspase-3 was further confirmed by poly(ADP-Ribose) polymerase (PARP) cleavage, a typical feature of caspase-dependent apoptosis. PARP was cleaved by TRAIL alone at 4 h (Figure 4A), while in combination with SH122, cleaved PARP survived up to 8 h. These results suggest that SH122 enhances TRAIL-induced apoptosis by activating caspases and promoting PARP cleavage.

To determine the combination effect of TRAIL and SH122 on the mitochondrial pathway, caspase-9 and Bid activation were examined. As shown in Figure 4B, Bid levels did not change at 4 h or 6 h, while at 8 h, Bid levels decreased after combination treatment, but not from treatment by TRAIL alone. Furthermore, caspase-9 cleavage could be detected as early as 4 h by either TRAIL alone or in combination with SH122, as evidenced by the appearance of its cleaved forms, p35 and p17. Interestingly, both pro- and cleaved caspase-9 were diminished at 8 h, indicating that caspase-9 activation was an early event with Bid cleavage (Figure 4B). These data suggest that the mitochondria pathway is also involved in TRAIL-induced, SH122-mediated apoptosis. Taken together, these *in vitro *apoptosis data reveal that SH122 potentiates TRAIL-induced apoptosis by activating both the extrinsic death receptor pathway and the intrinsic mitochondrial pathway, typically through activation of the caspase cascade. More importantly, SH-122 combined with TRAIL induced a longer-lasting apoptosis than TRAIL alone (8 h *vs. *4 h), showing that SH-122 enhanced the effect of TRAIL treatment.

### Downregulation of XIAP and cIAP-1 sensitizes TRAIL-induced cell death

**Figure 5:**
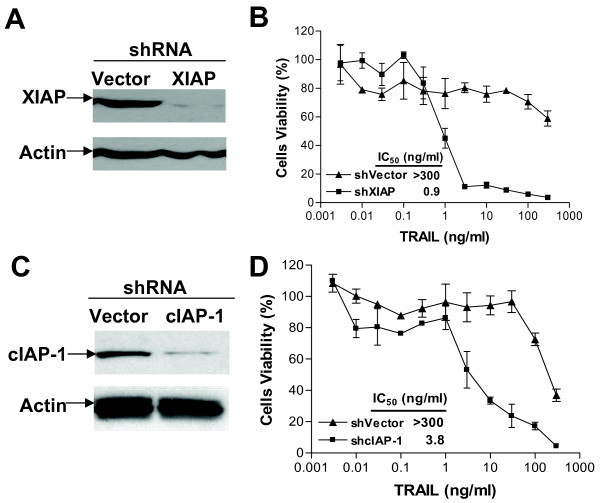
**Downregulation of XIAP or cIAP-1 sensitized TRAIL-induced cell death**. DU145 cells were transfected with shRNA of XIAP (shXIAP) (**A, B**) or cIAP-1 (shcIAP-1) (**C, D**), or the vector control (shVector), and knockdown effect was measured 48 h after transfection (**A, C**). Transfected cells were treated with TRAIL, and cytotoxicity was determined by CCK-8 detection kit (**B, D**). Normalized data were presented as mean ± SD (*n *= 3).

It is well established that IAPs (especially XIAP) are responsible for blocking TRAIL-induced apoptosis by inhibiting caspase functions [11,14,43]. To investigate the potential link between IAPs and TRAIL-resistance, short-hairpin RNA (shRNA) of XIAP or cIAP-1 was transfected into DU145 cells, and cytotoxicity was examined. As shown in Figure 5, XIAP or cIAP-1 knockdown shifted the cytotoxicity curve to the left, i.e., sensitized the cells to TRAIL. Based on IC_50_, a more than 300-fold sensitization was observed in the cells transfected with XIAP shRNA as compared with the vector control (Figure 5A and 5B). Similarly, in cIAP-1-shRNA transfected cells, an approximately 100-fold sensitization was achieved as compared with that of the vector control (Figure 5C and 5D). These results demonstrate that knockdown of XIAP and cIAP-1 effectively sensitizes the cells to TRAIL, indicating that both XIAP and cIAP-1 are involved in TRAIL-resistance, and that silencing XIAP and/or c-IAP1 can overcome such resistance in prostate cancer cells.

### Downregulation of XIAP attenuates sensitization potential of SH122 on TRAIL-induced cell death

**Figure 6:**
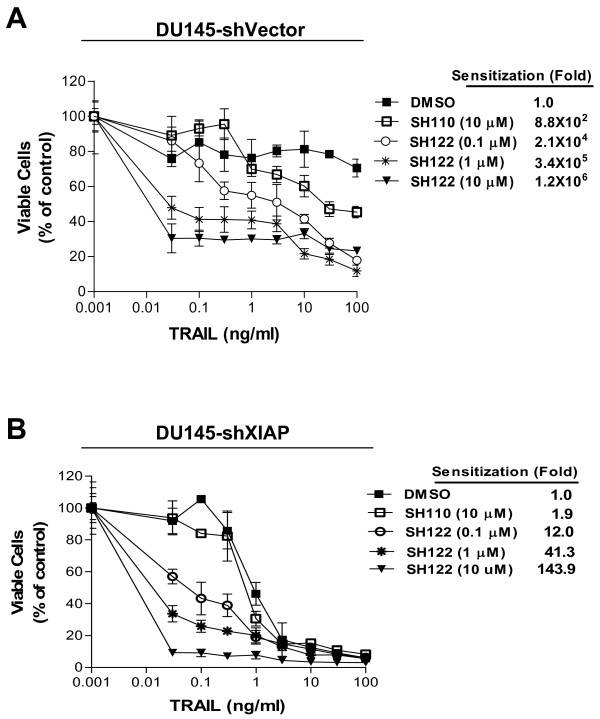
**Downregulation of XIAP attenuated sensitization effect of SH122 on TRAIL-induced cell death**. Cells transfected with either XIAP shRNA (**A**) or the vector control shRNA (**B**) were treated with serial diluted TRAIL, alone or in combination with 0.1, 1 and 10 μM of SH122, respectively, with 10 μM of SH110 as a negative control. Cytotoxicity was determined by CCK-8 detection kit. Normalized data were presented as mean ± SD (*n *= 3). Fold of sensitization was calculated by dividing IC_50 _of the compound-treated group by that of DMSO control.

Earlier studies have shown that both XIAP and cIAP-1 are specific targets of SH122 (Figure 1B and 1C), and knockdown of XIAP/cIAP-1 resulted in significant TRAIL-sensitization (Figure 5). To evaluate the role of XIAP in SH122-mediated TRAIL sensitization, we treated XIAP knockdown cells with SH122 and TRAIL. In vector shRNA (shControl)-transfected cells, negative compound SH110 showed a moderate sensitizing effect, while SH122 dramatically synergized TRAIL-induced cell death in a dose-dependent manner. Even at the lowest concentration (0.1 μM), SH122 was 22-fold more potent in sensitizing TRAIL than a high concentration of SH110 (10 μM) (Figure 6A). However, in XIAP shRNA-transfected cells, the sensitizing potential of SH122 was 10^2^~10^4^-fold less potent than shown by the shControl cells (Figure 6B). These results demonstrate that silencing XIAP dramatically attenuates SH122-mediated sensitization of TRAIL-induced cytotoxicity, suggesting that XIAP is an important target of IAP-inhibitors involved in TRAIL sensitization.

### SH122 inhibits TRAIL-induced NF-κB activation

**Figure 7:**
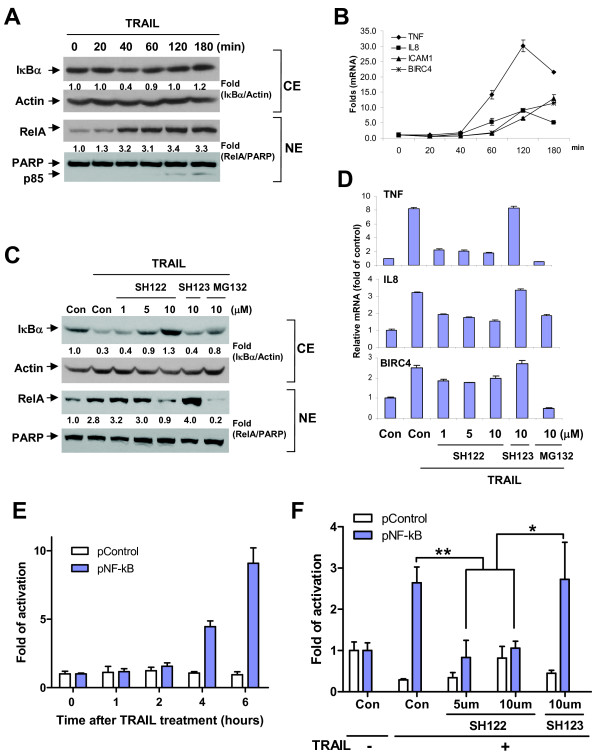
**SH122 suppressed TRAIL-induced NF-κB activation**. **A**. DU145 cells were treated with 300 ng/ml of TRAIL for the indicated time. Cytosol and nuclei subcompartments were fractionated for detection of IκBα and RelA, with Actin and PARP used as markers of cytosolic and nuclear extracts, respectively. Relative expression of IκBα and RelA was shown by dividing band intensity by that of Actin and PARP, respectively. CE, cytosolic extract; NE, nuclear extract. **B**. After treatment with 300 ng/ml of TRAIL, cells were harvested for quantitative RT-PCR detect NF-κB target genes. Fold increase of gene expression was calculated by dividing the normalized gene expression activity by that of the untreated control. **C**. Cells were pre-treated with desired compounds for 1 h, and challenged with TRAIL (300 ng/ml) for 40 min. Cytosol and nuclei subcellular compartments were fractionated for detection of IκBα and RelA, respectively. MG132 was used as a positive control for blocking NF-κB. **D**. After treatment as described in **C**, cells were harvested for quantitative RT-PCR to detect three NF-κB target genes. **E**, Time-course of TRAIL-induced NF-κB activation examined by NF-κB luciferase reporter assay. DU145 cells were transiently co-transfected with pNF-κB or pControl together with β-galactosidase plasmid, and then treated with TRAIL for the indicated time. Luciferase and β-galactosidase activities were measured as described in *Materials and Methods*. **F**, SH122 inhibited TRAIL-induced NF-κB activation in NF-κB luciferase reporter assay. Transfected DU145 cells were pretreated with SH122 or SH123 for 1 h followed by TRAIL treatment for 4 h. Fold of NF-κB activation was calculated by dividing the normalized luciferase activity by that of the untreated control. Representative results of at least two independent experiments. *Columns*, mean; *bars*, SD (*n *= 3). Con, DMSO vehicle control. ** P < 0.01; * P < 0.05, Student's t-test (*n *= 3).

Because NF-κB activation is known to play a crucial role in inhibiting apoptosis [19,21], we thought it was important to evaluate the effect of SH122 on NF-κB activation induced by TRAIL. First, we treated cells with TRAIL alone at the concentration that achieved apoptosis, to create optimal conditions for NF-κB activation in our system. TRAIL induced IκBα degradation by 60% at 40 min post-treatment, and concomitantly, nuclear RelA expression increased over 3-fold at 40 min and lasted for 3 h of treatment (Figure 7A). This revealed that TRAIL induced NF-κB activation via the classic pathway by degrading cytosolic IκBα and promoting RelA nuclear translocation. It is worth noting that at 120 min post treatment, cleaved PARP was observed in the nuclei extracts, suggesting a quick apoptosis induced by TRAIL along with NF-κB activation, also reflecting a balance of cell death and cell survival triggered by the same stimuli (Figure 7A). TRAIL consistently induced multiple NF-κB target genes expression. For the four target genes examined, TNF and IL8 expression reached their peak at 2 h post treatment, while ICAM-1 and BIRC4 expression continued to increase during treatment (Figure 7B).

Next, we pretreated DU145 cells with varying concentrations of SH122 or SH123 to evaluate the effect of the IAP-antagonist on TRAIL-induced NF-κB activation. Pre-treatment of SH122 potently suppressed IκBα degradation and RelA translocation in a dose-dependent manner (Figure 7C). At the same time point, SH122 was consistently shown to suppress expression of all three NF-κB target genes by 30-80% (*P *< 0.01 vs. control) even at a lower concentration (Figure 7D). In comparison, even high concentrations of the negative compound SH123 altered neither NF-κB protein redistribution (Figure 7C) nor NF-κB target genes expression (Figure 7D).

To further validate that SH-122 inhibits NF-κB pathway, we analyzed the effects of SH122 on NF-κB activation induced by TRAIL in DU145 cells using luciferase-based NF-κB reporter assay. We first examined the time-course of TRAIL-induced NF-κB activation by NF-κB reporter assay. 4 h TRAIL treatment resulted in ~5-fold NF-κB activation, and at 6 h over 9-fold activation was observed (Figure 7E), but at this time point the cells started to show signs of cell death. Therefore, we selected the 4 h TRAIL treatment in our NF-κB reporter assay. As shown in Figure 7F, SH122 inhibited TRAIL-induced NF-κB activation by >60% at doses of 5 and 10 uM, whereas the control compound SH123 had no such effect. Similar results were also observed in DU145 cells stimulated with TNFα (data not shown). These results demonstrate that SH122 exhibits a promising effect of blocking the TRAIL-induced NF-κB activation.

## Discussion

**Figure 8:**
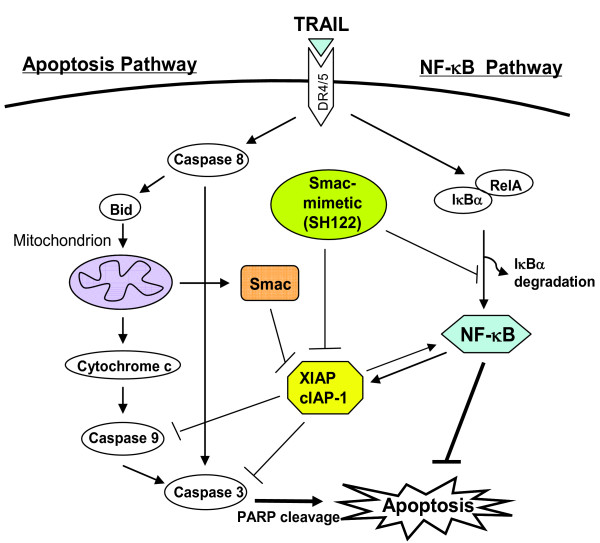
**Working model of Smac-mimetic IAP-antagonist sensitizing TRAIL-induced apoptosis by suppressing NF-κB**. TRAIL triggers apoptosis via both the death-receptor (DR4/DR5) and mitochondrial pathways, by activating initiator caspase-8 and -9, and effector caspase-3. Furthermore, both Bid and PARP are cleaved by caspases, which are typical predictors of TRAIL-mediated apoptosis. A Smac-mimetic effectively blocks IAP (XIAP/cIAP-1) function and facilitates caspase activation. Simultaneously, the Smac-mimetic suppresses TRAIL-induced classical NF-κB activation by preventing IκBα degradation and RelA nuclear translocation. Blockade of NF-κB - XIAP signaling by small molecule Smac-mimetic abolishes counteraction of pro-survival factors on TRAIL-mediated apoptosis.

In this study, we found that the small molecule Smac-mimetic SH122 potently sensitized TRAIL-induced apoptosis in multiple human prostate cancer cell lines. We also found that although downregulation of either XIAP or cIAP-1 sensitized TRAIL-mediated cytotoxicity, XIAP knockdown attenuated SH122-mediated TRAIL sensitization. Our results demonstrate that IAPs are valid molecular targets for modulating TRAIL sensitivity in prostate cancer cells, and show that blocking IAPs achieves improved efficacy and overcomes resistance to TRAIL. In addition, our results demonstrate that NF-κB is involved in regulating sensitivity of prostate cancer cells to TRAIL, and a Smac-mimetic can augment TRAIL-induced apoptosis by blocking both IAPs and NF-κB (Figure 8).

Smac-mimetic IAP-antagonists sensitize TRAIL-induced apoptosis by blocking XIAP function in multiple tumor models, including breast cancer [32], multiple myeloma [16], glioblastoma [11] and ovarian cancer [33]. Embelin, a natural XIAP inhibitor [44], sensitized TRAIL-induced apoptosis in pancreatic cancer cells [45]. These findings provide a strong rationale for using Smac-mimetics to achieve TRAIL-sensitization by functional inhibition of the overexpressed XIAP. To our knowledge, only a limited number of studies have reported on the combination of small molecule Smac-mimetic candidates with TRAIL in prostate cancer therapy. Interestingly, in prostate cancer cell lines, no strict correlation has been observed between XIAP expression and TRAIL responsiveness [3]. This discrepancy indicates that XIAP is not the only determinant of TRAIL-resistance in prostate cancer. Nevertheless, blocking XIAP function by transient overexpression of Smac achieved a promising enhanced efficacy in combination with TRAIL in prostate cancer cell lines [46], indicating that XIAP is the predominant target for TRAIL sensitivity. In our system, we found that the IAP-antagonist alone did not induce apoptosis [35], but potently sensitized TRAIL-induced cytotoxicity in TRAIL-resistant DU145 and LNCaP cells, as well as in relatively TRAIL-sensitive CL1 cells. Our finding, consistent with other reports [17,47,48], suggests that the IAP-antagonist exerts a potent sensitization effect independent of cell responsiveness to TRAIL, and provides an attractive strategy for using IAP-antagonists in combination treatment.

In addition to IAPs, NF-κB is another well documented pro-survival factor that is involved in mediating resistance to TRAIL-induced apoptosis in tumor cells [49]. It has been reported that constitutively active NF-κB signaling leads to TRAIL-resistance by upregulating XIAP in multiple human cancer cells [50], and in certain tumor cell types, NF-κB is the primary cause for TRAIL resistance [10]. Moreover, there is mounting evidence that cIAP-1 physically interacts with adaptor proteins in TNFα/TRAIL-mediated NF-κB signaling [51-53], suggesting that IAPs serve as "bridging" molecules between the apoptosis pathway and NF-κB pathway triggered by TRAIL (Figure 8). Thus it is reasonable to postulate that the pro-apoptotic IAP-antagonists may modulate NF-κB. Indeed, several recent studies reveal that Smac-mimetics (IAP-antagonists) can induce TNFα-dependent apoptosis in Smac-sensitive cell lines by degrading cIAPs and regulating NF-κB signaling [29-31]. These findings indicate that in cell lines that are sensitive to both Smac and TNFα, an IAP-antagonist itself is sufficient to induce cell death through autocrine TNFα signaling and caspase-8-dependent apoptosis [31,53].

Apart from these findings, our study shows that Smac-mimetics as a single agent induce neither cell death nor NF-κB activation in androgen-independent prostate cancer cells, suggesting that both apoptosis and NF-κB failed to be activated by the Smac-mimetic alone in chemo- or radioresistant cells with constitutively active NF-κB signaling. The mechanism underlying such a discrepancy in different cell types remains to be investigated. However, our data provide the first evidence that a potent Smac-mimetic IAP-antagonist directly blocks TRAIL-induced NF-κB activation in prostate cancer cells. In fact, at the concentration that effectively suppressed NF-κB activation, SH122 sensitized the effect of TRAIL several-hundred-fold, suggesting that blocking NF-κB by a Smac-mimetic is sufficient for TRAIL sensitization. Additionally, SH122-mediated inhibition of IκBα degradation reflects an effect at the level of the IκBα kinase (IKK) complex that is in agreement with others' observations in different systems [11,54], indicating activation of the canonical NF-κB pathway. Furthermore, SH122 suppresses XIAP mRNA expression driven by NF-κB, demonstrating that TRAIL-mediated sensitization by small molecule Smac-mimetic is associated with functional inhibition of both XIAP and NF-κB, especially in androgen-independent prostate cancer. Future work will focus on evaluating the response to TRAIL and the IAP-antagonists in androgen-dependent (AD) LNCaP cells and their androgen-independent (AI) derivative CL1 cells. This isogenic (for hormone dependence) cell model should permit us to determine if NF-κB plays an essential role in the transition from AD to AI prostate cancer and to discover if overcoming resistance to TRAIL-induced apoptosis can be achieved by down-modulating the NF-κB - IAP signaling pathway.

## Conclusion

Resistance to chemo- or radiotherapy, which is often associated with recurrence after prior androgen deprivation therapy in human prostate cancer, remains a severe clinical problem [55]. TRAIL is currently being evaluated in Phase I/II clinical trials, alone or in combination with other therapies, for the treatment of prostate cancer [3,10]. Our study indicates that blockade of IAPs by a small molecule Smac-mimetic promotes TRAIL-induced apoptosis in prostate cancer cells via modulating both the apoptosis pathway and NF-κB pathway. As IAPs are key molecular targets for the development of cancer cell-selective therapeutics, our findings reveal a potential mechanism for a Smac-mimetic IAP-antagonist on TRAIL-mediated signaling, and suggest that modulating IAPs may contribute to enhanced TRAIL efficacy, especially in androgen-independent prostate cancer with high levels of IAPs and constitutively active NF-κB signaling. Therefore, small molecule Smac-mimetics that specifically target IAPs may yield a potential therapeutic benefit with TRAIL-based therapy in chemo- or radioresistant prostate cancer.

## Competing interests

LX is a co-inventor of the Smac-mimetic compounds involved in the study. All other authors declare no competing interests.

## Authors' contributions

YD and ML contributed equally to this study. YD participated in the design of the study, performed NF-κB assay and drafted the manuscript; ML performed the cell culture, MTT assay, apoptosis assay, transfection and Western Blot, WT helped with the Western Blot analysis; YL and JL carried out NF-κB luciferase reporter assay; TSL participated in the project design and in revising the manuscript; LX supervised the study, experimental design, data analysis, and revision of the manuscript. All authors read and approved the final manuscript.

## Pre-publication history

The pre-publication history for this paper can be accessed here:

http://www.biomedcentral.com/1471-2407/9/392/prepub
